# Self-reported fever, treatment actions and malaria infection prevalence in the northern states of Sudan

**DOI:** 10.1186/1475-2875-10-128

**Published:** 2011-05-15

**Authors:** Khalid A Elmardi, Abdisalan M Noor, Sophie Githinji, Tareg M Abdelgadir, ElFatih M Malik, Robert W Snow

**Affiliations:** 1 National Malaria Control Programme, Federal Ministry of Health, P.O. Box 1204 Khartoum, Sudan; 2 Malaria Public Health & Epidemiology Group, Centre for Geographic Medicine Research - Coast, Kenya Medical Research Institute/Wellcome Trust Research Programme, P.O. Box 43640, 00100 GPO, Nairobi, Kenya; 3 Centre for Tropical Medicine, Nuffield Department of Clinical Medicine, University of Oxford, CCVTM, Oxford OX3 7LJ, UK; 4 Directorate General of Preventive Medicine and PHC, Federal Ministry of Health, P.O. Box 1204, Khartoum, Sudan

## Abstract

**Background:**

The epidemiology of fevers and their management in areas of low malaria transmission in Africa is not well understood. The characteristics of fever, its treatment and association with infection prevalence from a national household sample survey in the northern states of Sudan, an area that represents historically low parasite prevalence, are examined in this study.

**Methods:**

In October-November 2009, a cluster sample cross-sectional household malaria indicator survey was undertaken in the 15 northern states of the Sudan. Data on household assets and individual level information on age, sex, whether the individual had a fever in the last 14 days and on the day of survey, actions taken to treat the fever including diagnostic services and drugs used and their sources were collected. Consenting household members were asked to provide a finger-prick blood sample and examined for malaria parasitaemia using a rapid diagnostic test (RDT). All proportions and odds ratios were weighted and adjusted for clustering.

**Results:**

Of 26,471 respondents 19% (n = 5,299) reported a history of fever within the last two weeks prior to the survey and 8% had fever on the day of the survey. Only 39% (n = 2,035) of individuals with fever in last two weeks took any action, of which 43% (n = 875) were treated with anti-malarials. About 44% (n = 382) of malaria treatments were done using the nationally recommended first-line therapy artesunate+sulphadoxine-pryrimethamine (AS+SP) and 13% (n = 122) with non-recommended chloroquine or SP. Importantly 33.9% (n = 296) of all malaria treatments included artemether monotherapy, which is internationally banned for the treatment of uncomplicated malaria. About 53% of fevers had some form of parasitological diagnosis before treatment. On the day of survey, 21,988 individuals provided a finger-prick blood sample and only 1.8% were found positive for *Plasmodium falciparum*. Infection prevalence was higher among individuals who had fever in the last two weeks (OR = 3.4; 95%CI = 2.6 - 4.4, p < 0.001) or reported fever on the day of survey (OR = 6.2; 95%CI = 4.4 - 8.7, p < 0.001) compared to those without a history of fever.

**Conclusion:**

Across the northern states of the Sudan, the period prevalence of fever is low. The proportion of fevers that are likely to be malaria is very low. Consequently, parasitological diagnosis of all fevers before treatment is an appropriate strategy for malaria case-management. Improved regulation and supervision of health workers is required to increase the use of diagnostics and remove the practice of prescribing artemisinin monotherapy.

## Background

The diagnosis and treatment of fevers in Africa is undergoing a paradigm shift, away from the widely promoted concept that all fevers should be regarded as malaria [1-6] to more informed guidelines that promote the use of parasitological diagnostics and a rational use of artemisinin-based combination therapies [7]. This migration from presumptive to parasitological confirmed diagnosis is for a combination of reasons: the costs of replacement therapies for failed monotherapy [8], the recognition that parts of Africa are undergoing an epidemiological transition that supports *Plasmodium falciparum *transmission intensities considerably lower than ten years previously with a natural consequence that most fevers are not associated with malaria infection [9-11] and a more general ambition to improve the standards and quality of malaria case-management and its corollary that non-malaria febrile conditions receive better investigation, intervention and prognosis, and the advent of rapid diagnostic tests [7,12-15].

However, the understanding of the epidemiology of fevers and their management in areas where parasite exposure is infrequent is limited. Research into the epidemiology of malaria and understanding the impacts of control and disease management have traditionally focused on stable, high transmission areas of Africa. This study investigates the characteristics of fever management and infection prevalence from a national household survey in the northern states of Sudan, an area of historically low malaria risk.

## Methods

### Malaria in the northern states of Sudan

Over 27 million people live in the 15 northern states of Sudan with the National Malaria Control Programme based in Khartoum responsible for the implementation of federal policies and guidelines for malaria control. Stable, malaria transmission to both *P. falciparum *and *Plasmodium vivax *has historically been constrained by the arid climate among the most northern states that border Egypt [16-18]. In 2007, it was estimated that 5.7 million people were exposed to stable *falciparum *and to limited *vivax *malaria transmission in the northern states of Sudan [19,20], with a highly over-distributed pattern of risk that was generally characterized by low parasite prevalence [19,21]. In 2005, a household sample survey reported an overall *P. falciparum *infection prevalence of 3.7% among all age groups using microscopy and a 14-day period prevalence of self-reported fever of 37% in eight northern states [21]. Despite the low prevalence of malaria infection it has been estimated that over 50% of all fevers are managed as malaria [22,23]. By 2006, malaria was thought to contribute to 50% of all out-patient diagnoses reported as part of the Health Information System of the northern states of the Sudan and accounted for an estimated 44,000 deaths in 2002 [18].

Against a background of escalating chloroquine resistant *P. falciparum *[24,25], northern states of the Sudan changed their treatment policy to artemisinin-based combination therapy (ACT) in June 2004 [26]. The current standard treatment protocol for uncomplicated falciparum malaria is artesunate plus sulphadoxine/pyrimethamine (AS+SP), as first-line and artemether-lumefantrine (AL) as second-line of treatment [23,27]. Overseas development assistance to support scaling up of malaria control activities rose sharply in 2004 with support from the Global Fund for AIDS, TB and Malaria (GFATM) approved malaria funding and by 2009 approximately $33 million had been disbursed by the GFATM [28] to support malaria activities across the northern states of the Sudan. Since 2005, the Sudan national malaria control programme (NMCP) has distributed over 6 million long-lasting insecticide treated nets, expanded residual insecticide spraying of houses in selected areas; and supported the improvement of malaria case-management through in-service training of health workers in revised standard treatment guidelines and increased use of diagnostics and improving the supply of AS+SP to over 90% of government health facilities.

### Household malaria survey

A household sample survey of basic malaria coverage indicators was undertaken between October and November 2009 across the northern 15 states of Sudan. A sample of 6,000 households across 300 clusters was selected using a multi-stage probability proportional to size (PPS) cluster sample design. The number of households required was determined using the Roll Back Malaria Monitoring and Evaluation Reference Group for indicators in young children (RBM-MERG) approach [29] with survey powered to detect changes in the proportion of children under the age of five years sleeping under an ITN with precision at the national and state levels. As a compromise between precision and logistics, an average of 20 households per clusters was used to estimate the number of sampling units to survey. To identify the location of the clusters to be surveyed a list of Popular Administrative Units (PAUs) from the National Statistics and Census Office (NSCO) was used to determine the PAUs in which survey clusters would be located. The PAUs in each state were selected according to PPS method distributed proportionately into urban and rural within each state as defined during the 2008 national census. The list and size of all enumeration areas (EAs) within the selected PAU was then assembled by the NSCO and a further sampling to select the clusters (EAs) to be surveyed was undertaken using the PPS method separately for urban and rural. Within each cluster, 20 households were randomly selected and all members of the households visited at the time of survey were interviewed using the relevant survey questionnaire.

A community sensitization exercise was undertaken a few weeks before and throughout the survey period in the print, radio and television media by the NMCP of the Federal Ministry of Health. Thirty coordinators selected at national and state levels were trained to be in-charge of the day-to-day management of the survey in their respective states. A total of 196 field team members (28 teams) were trained on general interviewing skills, administration of consent forms, filling of questionnaires, collection of blood samples, and appropriate treatment of individuals found positive for malaria. The team members were also trained in the use of global positioning systems for mapping of clusters. Each of the 28 survey teams, consisting of seven persons (one supervisor; two interviewers; two nurses or equivalent; and two laboratory technicians) visited one cluster per day.

A household members' questionnaire was adapted from the standard RBM-MERG tools [29] to ensure information was collected from all age groups. The questionnaire was developed first in English and translated into Arabic and draft versions were checked and piloted independently by members of the survey teams conversant in both languages to reduce ambiguities in translation. This questionnaire captured information on each usual and visiting resident member at household during the survey. Information including: age, sex, educational status, relationship to the head of the household, use of mosquito nets and insecticide-treated nets (ITN) the night prior to the survey, whether the room slept in last night had been subject to residual spraying in the last 12 months, and whether the individual had had a fever in the last 14 days were captured. Additional information was collected on whether the fever was present on the day of the survey, actions taken to treat the fever including diagnostic services used, drugs used and their sources. At the end of each interview each household member was asked to provide a finger prick blood sample following informed consent and examined for malaria parasitaemia using an RDT (First Response Malaria Ag (pLDH/HRP2) COMBO, Premier Medical Corporation Ltd). The test gave results on whether infection was due to *P. falciparum *or mixed species but did not distinguish between *P. falciparum *and *P. vivax*. At the end of each survey day, all questionnaires and RDTs were submitted to the state coordinators for review and storage. The state coordinators reviewed the survey team's daily submissions and suggested corrections where necessary.

### Data entry and analysis

Twenty trained data entry personnel were hired to capture information from the survey questionnaires using customized data entry screens developed in Microsoft Access 2007. Once entered, data were checked for consistencies by a data manager and correctable errors reconciled.

Reports of fever were classified into two categories; those who reported to have had a fever in the last two weeks preceding the survey and those who reported fever present on the survey day. Treatment actions and drug classes were classified as first actions only. Common across all malaria indicator surveys, discerning multiple treatment sources, their sequence and timing has proved complex and difficult to interpret. Where surveys have investigated multiple treatment actions the first action is usually the dominant reported action and second or third actions are relatively rarely reported [30,31]. Descriptive analysis of fever and infection prevalence was undertaken according to age, sex, rural-urban residence, use of ITN and wealth quintile. All data were analyzed using STATA version 11.0 (StatCorp, College Station, Texas). All proportions and odds ratios were weighted and adjusted for clustering using the *svy: *command in STATA. Because the selection of the PAUs and the clusters was done probability proportional to size, sample weighting was implemented only at the household level and weights were derived as the inverse of the probability of selecting a household within a cluster. Multivariate regression models were fitted using the *svy: logistic *command allowing for clustering and weighting of the data to assess the association of fever as a risk factor for malaria infection. The analysis controlled this association for age, residence, use of ITN, and wealth quintile. For each predictor the Odds Ratio (OR), 95% confidence interval (CI), and P-values were recorded.

### Ethical considerations

Ethical clearance was provided by the National Research Ethics Committee of the Federal Ministry of Health (fmoh/rd/SEC/09, date: 7/9/2009). All household members and parents/guardians fo children were informed about all relevant aspects of the study including its aim, procedures, potential risk and hazards. A signed informed consent was obtained and assurance given that the participation in the study was strictly voluntary and all information would remain confidential and anonymized. Persons who volunteered a blood sample for RDT malaria testing and were found positive were treated immediately by survey nurses using nationally recommended AS+SP age-defined dosage.

## Results

### Survey characteristics

A total of 5,980 of 6,000 sampled households were surveyed from 300 clusters. 26,471 individuals from the households were available for interview during the survey. 55.7% of surveyed individuals were female, 19.4% were children under five years of age and 64.3% were from rural areas. About 83% (n = 21,988) of the individuals provided a finger prick blood sample for testing malaria parasites while the rest refused to provide blood samples following informed consent and requests for voluntary participation.

### Epidemiological features of reported fever and infection prevalence

Among all surveyed individuals, 19% reported a history of fever in the last two weeks preceding the survey date and 8% were febrile on the day of survey (Table 1, Additional File 1). There were very small differences with age, however female and rural residents had higher fever prevalence (in the last two weeks or on the day of survey) compared to male and urban residents respectively (Table 1). Fever prevalence was more than two times higher among individuals from the poorest household compared to those from the least poor households. Among the 21,988 individuals who volunteered a blood sample for RDT examination for the presence of malaria infection only 1.8% were found positive (Table 2, Additional File 1). It is assumed that the majority of these infections were *P. falciparum *however it is not possible to disaggregate vivax or other Plasmodium infections from the reported positive cases. There was minimal age pattern in infection prevalence (Table 2). The prevalence of infection was higher in rural areas (2.4%) compared to urban residents (0.8%); among those who did not use nets (2.4%) compared to those who did (1.6%) and among individuals from the poorest households (2.7%) compared to the least poor (2.7%). Importantly, the multivariate regression showed that after adjusting for gender, age, residence, use of ITN and wealth quintile, individuals who reported fever in the last two weeks and on the day of survey were three times (Odds Ratio (95% confidence interval) 3.4 (2.6-4.4)) and 6 times (OR (95% CI) 6.2 (4.4-8.7)) more likely to have malaria infection (Table 2). Interestingly, use of ITN was not significantly associated with the likelihood of having malaria infection.

**Table 1:** Prevalence of fever in last two weeks, on the day of survey and treatment seeking (N = 26,471) and malaria infection tested using rapid diagnostic tests (N = 21,988) by gender, age, residence, used of insecticide treated nets (ITN) and wealth quintile.

	Number of persons interviewed	Fever			Malaria infection
		
		Fever last 2 weeks % (95% CI) (n/N = 5,299/26471)	Fever on survey day % (95% CI) (n/N = 2,370/2,6471)	Fevers for which treatment was sought (n/N = 2,035/5,299)	Positive % (95% CI) (n/N = 489/21,988) *2
**Gender**					
Male	11,720	16.8 (15.6-17.9)	7.3 (6.4-8.1)	42.6 (39.2-46.0)	2.1 (1.6-2.7)
Female	14,751	20.9 (19.6-22.2)	9.1 (8.3-10.0)	36.7 (34.2-39.2)	1.5 (1.1-2.0
*^1^**Age**					
<1	1,148	20.7 (18.3-23.3)	11.0 (8.9-13.2)	45.2 (37.7-52.6)	2.1 (1.1-3.2)
1-4	3,982	23.3 (21.3-25.3)	7.8 (6.6-9.0)	44.2 (40.1-48.2)	2.0 (1.2-2.9)
5-9	4,145	15.9 (14.3-17.5)	7.1 (6.0-8.2)	40.2 (35.0-45.3)	3.0 (2.0-4.0)
10-19	5,432	14.5 (13.1-15.8)	5.9 (5.0-6.9)	37.0 (32.6-41.4)	2.1 (1.4-2.8)
>19	11,764	20.6 (19.4-21.8)	9.7 (8.9-10.6)	37.0 (34.4-39.6)	1.1(0.8-1.4)
**Residence**					
Urban	9459	16.1 (14.3-17.9)	6.9 (5.6-8.2)	46.1 (42.6-49.6)	0.8 (0.5-1.1)
Rural	17,012	21.2 (19.5-22.9)	9.4 (8.3-10.5)	35.0 (31.7-38.2)	2.4 (1.7-3.2)
**Use of ITN**					
No	23,377	18.6 (17.5-19.8)	7.9 (7.2-8.7)	38.9 (36.3-41.6)	2.4 (1.2-3.6)
Yes	3,094	22.2 (20.0-24.5)	11.1 (9.2-13.1)	39.5 (34.7-44.4)	1.6 (1.2-2.1)
**Wealth quintile**					
Least Poor	5,649	13.6 (12.0-15.3)	4.3 (3.3-5.2)	53.0 (48.6-57.4)	0.4 (0.2-0.7)
Second	5,776	14.9 (13.3-16.4)	5.9 (4.8-7.1)	44.2 (39.5-48.9)	1.1 (0.7-1.6)
Third	5,183	21.4 (19.2-23.7)	9.9 (8.3-11.6)	40.6 (36.4-44.8)	1.9 (1.1-2.6)
Fourth	5,006	22.3 (20.0-24.8)	10.9 (9.2-12.7)	34.5 (30.4-38.5)	3.3 (1.7-4.9)
Most Poor	4,857	26.4 (23.3-29.5)	12.8 (10.8-14.7)	27.2 (22.8-31.6)	2.7 (1.5-4.0)
**Total**	**26,471**	**19.1 (17.9-20.2)**	**8.3 (7.6-9.1)**	**39.1 (36.6-41.5)**	**1.8 (1.3-2.2)**

Proportions are weighted and cluster adjusted.

*^1 ^67 individuals did not have age data

*2 Of the 4,483 individuals who did not provide finger-prick sample for malaria testing, 51% were urban, 52% were male, with refusal highest in Khartoum state at 25%.

**Table 2:** Results of multivariate regression analysis of the association of fever with malaria when controlled for gender, age, residence, use of insecticide treated nets (ITN) and wealth quintile.

	Fever two weeks included: Odds Ratio (95% CI), p-value	Fever on the day of survey included: Odds Ratio (95% CI), p-value
**Fever in the last two weeks**		
No	Ref.	-
Yes	3.4 (2.6-4.4), <0.001	-
**Fever on the day of survey**		
No	-	Ref.
Yes	-	6.2 (4.4-8.7), <0.001
**Gender**		
Male	Ref.	Ref.
Female	0.7 (0.6-0.9), 0.013	0.7 (0.6-0.9), 0.017
*^1^**Age**		
<1	Ref.	Ref.
1-4	1.0 (0.5-1.8), 0.935	1.1 (0.6-2.1), 0.723
5-9	1.7 (0.9-3.1), 0.074	1.8 (0.9-3.1), 0.054
10-19	1.4 (0.8-2.7), 0.255	1.6 (0.8-2.9), 0.173
>19	0.6 (0.4-1.2), 0.150	0.7 (0.4-1.2), 0.143
**Residence**		
Urban	Ref.	Ref.
Rural	1.7 (1.1-2.9), 0.037	1.9 (1.1-3.2), 0.022
**Use of ITN**		
No	Ref.	Ref.
Yes	1.4 (0.8-2.2), 0.201	1.4 (0.8-2.2), 0.221
**Wealth quintile**		
Least Poor	Ref.	Ref.
Second	2.2 (1.2-4.1), 0.015	2.0 (1.1 -3.8), 0.031
Third	3.0 (1.6-5.7), 0.001	2.7 (1.4-5.1), 0.003
Fourth	4.6 (2.3-9.5), <0.001	4.0 (1.9-8.5), <0.001
Most Poor	3.4 (1.6-7.5), <0.002	2.9 (1.4-6.5), 0.007

*^1 ^14 individuals did not have age data; Ref. = reference.

### Treatment actions for fever, access to diagnostic facilities and associations with infection prevalence

Among the 5,299 self-reported fevers occurring during the 14 days prior to the survey 39% (n = 2,035) had some treatment action (Table 1). Treatment seeking was higher among female, children, urban residents and those from the least poor households. Among those who sought treatment, 95% were treated with 'Western-type' drugs, 43% were treated with anti-malarials while the remaining 57% were treated with other drugs, mainly antipyretics (Table 3, Additional File 1). Almost 53% of those febrile individuals who sought treatment were tested (Table 3) with testing rates higher among urban residents (70%) compared to rural (42%). Majority of treatments and tests were undertaken in government health facilities, mainly health centres and hospitals and only about 22% of all treatments were in the private sector. Among the 875 individuals who were treated for malaria, 44% were treated with the nationally recommended first-line therapy for uncomplicated malaria, AS+SP with 87% of these treatments occurring in the government sector (Table 4, Additional File 1). Chloroquine or SP, monotherapies not recommended for the management of malaria in the northern states of Sudan, were used to treat 13% of all malaria cases and quinine was reportedly administered for 5.0% (Table 4). Importantly 34% of all malaria treatments were reportedly managed using injectable artemether monotherapy which was more frequently obtained from public sector facilities, notably from higher order facilities such as hospitals and health centres (Table 4).

**Table 3:** Type of treatment and prevalence parasitological diagnosis before treatment by source among individuals who reported having fever in last two weeks and who took action (N = 2,035).

	Use of any drug, % (95% CI) (n/N = 1,935/2,035)	Use of anti-malarials, % (95% CI) (n/N = 875/2,025)	Use of other drugs, % (95% CI) (n/N = 1,060/2,035)	Parasitologically tested for malaria before any treatment, % (95% CI) (n = 1,015/2,035)*
**Overall**	95.4 (94.4-96.4)	42.6 (39.2-46.0)	57.4 (54.0-60.8)	52.8 (48.6-57.0)

**By treatment source**				
**Government**				
Hospital	29.0 (25.1-32.9)	28.2 (23.1-33.4)	29.6 (25.4-33.7)	38.8 (33.1-44.6)
Health centre	30.3 (26.4-34.3)	33.7 (28.2-39.2)	27.7 (23.7-31.7)	36.9 (31.4-42.4)
Basic health unit	11.4 (8.1-14.5)	11.4 (7.4-15.5)	11.3 (8.1-14.6)	4.6 (2.8-6.2)
Community Health Worker	6.9 (4.5-9.3)	5.9 (3.2-8.6)	7.7 (4.9-10.6)	3.4 (1.9-5.0)

**Private**				
Health facility/pharmacy/drug Store	15.3 (12.8-17.7)	16.0 (12.3-19.7)	14.7 (12.0-17.5)	15.2 (11.6-18.9)
Shop	5.5 (4.0-6.9)	3.8 (2.2-5.3)	6.8 (4.8-8.8)	0.4 (0.0-0.9)
Other	1.6 (0.9-2.3)	9.5 (0.2-1.7)	2.2 (1.0-3.3)	0.5 (0.0-1.1)

Proportions are weighted and cluster adjusted.

*Individuals tested for malaria when reporting at a health facility with fever. 57% of these received anti-malarials compared to 29% of those who were not tested. It was not possible to discern whether only the positive cases were treated with anti-malarials because of problems with recall and incompleteness of test status.

**Table 4:** Type of anti-malarials used by source among individuals who reported having fever in last two weeks and who were treated for malaria (N = 875).

	Anti-malarial type				
	
	AS+SP (n = 382)	SP or Chloroquine (n = 122)	Quinine (n = 39)	Artemether (n = 296)	**Other **^† ^**(n = 36)**
**Overall**	43.9 (39.6-48.2)	13.4 (10.6-16.2)	4.5 (2.8-6.1)	33.7 (30.0-37.4)	4.6(2.7-6.5)

**By treatment source**					
**Government**					
Hospital	29.0 (21.2-36.8)	20.3 (11.3-29.3)	50.2 (27.8-72.6)	28.4 (21.4-35.3)	21.4 (7.6-35.1)
Health centre	36.5 (27.3-45.7)	24.0 (14.7-33.2)	33.6 (11.1-56.1)	35.8 (29.2-42.4)	20.2 (4.7-35.8)
Basic health unit	10.5 (4.9-16.0)	14.6 (6.6-22.7)	5.4 (-0.0-11.1)	13.7 (7.6-19.7)	
Community Health Worker	7.0 (2.1-11.8)	5.8 (0.7-11.0)	7.2 (-0.2-14.6)	4.4 (1.7-7.2)	5.5 (-5.0-15.9)
**Private**					
Health facility/pharmacy/drug Store	14.6 (9.3-20.0)	20.0 (10.9-29.1)	2.1 (-1.9-6.2)	14.7 (10.3-19.1)	40.2 (18.3-62.1)
Shop	1.6 (0.3-28.6)	15.2 (7.3-23.2)	1.4 (-1.4-4.2)	1.1 (-0.6-2.9)	12.7 (-4.3-29.9)
Other	0.7 (-0.0-1.7)			1.8 (-0.0-3.8)	

Proportions are weighted and cluster adjusted.

^†^13 individuals took AL, 23 took unspecified anti-malarial drugs;

Of those individuals who reported a fever episode in the last two weeks and took action 40% took action within 24 hours and 67% within 48% hours. Of those that were treated within 24/48 hours about 22% were treated with AS+SP, 16.1% with artemether, 2% with quinine, 5% with SP or CQ and about 55% with antipyretics and other non-anti-malarial drugs.

## Discussion

Infection prevalence, as judged by RDT positivity rates, among 21,988 people sampled across 300 communities of the 15 northern states Sudan was very low, 1.8%. These results have not been corrected using polymerase chain reaction detection, to account for the documented false positive rates [13,32] and it is possible that the true prevalence between October and November 2009 is lower. The prevalence documented across eight northern states in 2005 using microscopy was 3.7% and it is possible that prevalence might be declining in the northern states of Sudan. However, such comparisons should be made with caution because of the acutely seasonal nature of transmission in semi-arid areas and the limitations of point-estimates of transmission in such areas. Nevertheless against a background of low malaria infection risk, nearly one in five people reported a febrile event during the two-week period prior to the survey. It is not clear what the majority of these events represent in clinical and public health terms as almost 60% of febrile people do not seek any treatment and are apparently well during the survey interview. However 40% did seek treatment and almost half of these treatment actions included the use of anti-malarials. The prevalence of "malaria" treatment is higher than a point prevalence of infection and may suggest a degree of over-treatment. However, it is also important to note that the prevalence of infection is over six-times higher (OR = 6.2; Table 2) among patients with a fever on the day of the survey and in an area where transmission intensity is low and the acquisition of functional clinical immunity to new infections is low [33], infection may be more directly related to symptoms, as such the prevalence of fever and infection is closer to the reported anti-malarial use recorded during this survey. Clearly this presumes that those fevers that don't seek treatment are not associated with malaria and those who seek treatment are appropriately defined as malaria. Neither of these caveats can be tested directly through cross-sectional surveys.

When treatment for fevers was sought, over 70% of patients were managed at the Government's health facilities that are managed by State health authorities. The use of the private retail sector in the northern states of Sudan is not as prolific as described in many other African settings [34]. Partly as a consequence of the high government sector use, the most striking observation was the wide-spread reported access to parasitological diagnosis among febrile respondents who sought treatment (53%; Table 3). Strengthened laboratory support for malaria diagnosis has been an important part of the recent malaria case-management strategy [23]. It was not possible to stratify analysis on the reported test results as these data were incomplete and were not verified against patient or care-giver notes to distinguish true test positive results from the results told to the patients. However, what is clear is that parasitological testing *per se *did influence the prescriptions made and a higher proportion of tested patients received the nationally recommended first-line treatment compared for uncomplicated malaria to those not tested for malaria (data not shown). Separate studies are underway to examine the quality of care and prescription practices among health workers in the government health sector to investigate prescribers' adherence to parasitological test results and the associated prescriptions. The international milestones for successful implementation of case-management are currently anchored around 60%, 80% or universal treatment of all fevers with anti-malarial drugs within 24/48 hours [35-37]. The Sudan National Malaria Strategy for the northern states outlines the ambition that by 2012 "80% of malaria patients will receive prompt and effective treatment" [23]. In addition the current national malaria strategy aims at reaching 100% laboratory confirmation of reported malaria incidence by 2010 [23]. A more adequate target for Sudan and many countries in Africa is that all fevers are tested for malaria and all those reported as positive for malaria infection are treated with national first-line recommended therapy. That only 19% of fevers (and 45% of those who were treated with anti-malarials) were treated with nationally recommended AS+SP and that only 14% were managed with AS+SP within 48 hours is not a failure to reach established milestones as most fevers are not malaria and thus should not be held to account as a failure. Indeed it could be argued that the fewer fever/anti-malarial drug exposures the greater the success of programmes promoting effective diagnostic strategies.

Of greatest concern was the reported use of artemether, available in injectable forms at Government clinics, despite an international ban on its use to manage uncomplicated malaria by the World Health Organization in 2006 [38]. The use of artemether in Sudan has been attributed to a belief by some prescribers and patients that injections have a stronger effect and work faster than oral medications and that there may be patient pressure to use these medicines [39,40]. Furthermore parasitological diagnosis of malaria was a predictor of artemether use, where patients were almost 3 times more likely to receive this drug if tested compared to if no parasitological test was done (data not shown). Chloroquine and SP continue to be available at government clinics and are prescribed, more frequently if no parasitological diagnosis is attempted than when malaria testing is performed, despite the reported wide-spread resistance across Sudan to both these mono-therapies [22,41,42]. Non-adherence to national standard treatment guidelines at the point of care is common across many settings in Africa [43-47], previously reported in Sudan [48] and their reasons and determinants complex [45,49]. Exploring the prescription practices and patient treatment actions demands a more rigorous combination of facility and community-based qualitative and quantitative investigations than was possible through the large-scale single cross-sectional sample survey described here.

About 67% of fevers that sought treatment in the current study were treated after 48 hours of onset of symptoms. Similar findings have been reported in Sudan where 40% of the fever cases sought treatment after 72 hours of onset of symptoms and a mean delay of 67.8 hours before attending a health facility [22,50]. Lack of money, waiting for improvement, low coverage with health facilities, dissatisfaction with services, difficulty to reach the facilities especially during rainy season, and waiting for the effect of traditional remedies have been highlighted as some of the reasons for delayed treatment [26,51].

## Conclusions

This study demonstrates that fever prevalence in the Sudan is low and with a *P. falciparum *infection prevalence of only 1.8%, the proportion of fevers attributable to malaria would also be extremely low. Consequently, parasitological diagnosis of malaria at all levels of the health system must become part of the malaria case management policy. Although the current testing rate of fevers of 53% is higher than those of most African countries, it still remains low largely because microscopy in the Sudan is limited to hospitals and health centres [52] and RDTs have not been scaled up nationally. Ongoing revision of the national malaria strategy aims to commit the northern states of the Sudan to parasitological diagnosis of all fevers which will be supported with rapid scale-up of RDTs in the all public health facilities, which provide almost 80% of all fever treatments [Abdalla, Personal Communication]. Concerns remain, however, on the types of anti-malarials used for the treatment of fevers with both chloroquine and SP still in circulation, both of which have failed in the northern states of Sudan in the treatment of *P. falciparum *infections, although chloroquine is still used for the treatment of *P. vivax *malaria in the country. Importantly, Sudan must urgently focus attention on stopping the widespread use of artemether for fever treatment, a monotherapy banned internationally by the World Health Organization for treatment of uncomplicated malaria, to avoid the emergence of resistance of artemisinin which could lead to devastating consequences both in the Sudan and the rest of Africa. Interestingly, majority of patients who received artemether have obtained this drug from the public health sector and an investigation of the reasons behind this practice, given that the health workers from this sector have had extensive training in malaria case-management, should be undertaken.

## Abbreviations

ACT: artemisinin-based combination therapy; AS+SP: artesunate (AS) plus sulphadoxine/pyrimethamine (SP); GFATM: Global Fund to Fight AIDS, Tuberculosis and Malaria; LLIN: long lasting insecticide treated nets; MIS: Malaria Indicator Survey; NMCP: National Malaria Control Programme; RBM-MERG: Roll back Malaria-Monitoring and Evaluation Reference Group; WHO/EMRO: World Health Organization-Eastern Mediterranean Regional Office (WHO-EMRO); RDT: Rapid Diagnostic Test; PAU: Popular administrative unit; FMoH: Federal Ministry of Health.

## Competing interests

The authors declare that they have no competing interests.

## Authors' contributions

KAE was involved in design and development of survey tools, implementation of survey, and collection of data and drafting of the manuscript. AMN designed the study, collected, cleaned and analysed the data and participated in drafting the manuscript. SG reanalyzed the data and participated in drafting the manuscript. TMA was involved in design and development of survey tools, implementation of survey, and collection of data and drafting of the manuscript. EMM has participated in the design and planning of the study. He has revised the manuscript as well. RWS conceived the questions for this manuscript and provided direction on analysis and drafting the manuscript.

## Supplementary Material

Additional file 1 **A summary of numerators (n) and denominators (N) for Tables 1, 3 and 4 in the main text **The additional file presents the numbers of persons: interviewed during the 2009 national malaria indicator survey in the northern states of Sudan; with fever in the last two weeks and on the day of survey; sought treatment for fever; tested for malaria; positive for malaria; used drugs; used antimalarials; and by type of antimalarial. Click here for file
