# Supplementary material for: Self-reported fever, treatment actions and malaria infection prevalence in the northern states of Sudan
Source: Malar J. 2011 May 15;10:128. doi: 10.1186/1475-2875-10-128 (PMC3115918; doi:10.1186/1475-2875-10-128)
Supplement: Additional file 1 — A summary of numerators (n) and denominators (N) for Tables 1, 3 and 4 in the main text The additional file presents the numbers of persons: interviewed during the 2009 national malaria indicator survey in the northern states of Sudan; with fever in the last two weeks and on the day of survey; sought treatment for fever; tested for malaria; positive for malaria; used drugs; used antimalarials; and by type of antimalarial. [file 1475-2875-10-128-S1.DOC]

**Additional File 1: Self-reported fever, treatment actions and malaria infection prevalence in the northern states of Sudan**

**Table 1 Number of sampled individuals with fever in last two weeks and on the day of survey; those who sought treatment; and those who had malaria infection tested using rapid diagnostic tests by gender, age, residence, used of insecticide treated nets (ITN) and wealth quintile.**

|  | **Number of persons interviewed** | **Fever** | | | **Malaria infection** | |
| --- | --- | --- | --- | --- | --- | --- |
|  |  | **Fever last**  **2 weeks** | **Fever on**  **survey day** | **Fevers for which treatment was sought** | **Tested** | **Positive** |
|  |  |  |  |  |  |  |
| **Gender** |  |  |  |  |  |  |
| Male | 11,720 | 2,076 | 915 | 876 | 9,402 | 243 |
| Female | 14,751 | 3,223 | 1,455 | 1159 | 12,586 | 246 |
| ***^1^Age** |  |  |  |  |  |  |
| <1 | 1,148 | 242 | 135 | 137 | 830 | 20 |
| 1-4 | 3,982 | 950 | 330 | 524 | 3,268 | 83 |
| 5-9 | 4,145 | 713 | 320 | 433 | 3,447 | 131 |
| 10-19 | 5,432 | 829 | 341 | 532 | 4,515 | 126 |
| >19 | 11,764 | 2,547 | 1,234 | 1,632 | 9,870 | 125 |
| **Residence** |  |  |  |  |  |  |
| Urban | 9459 | 1,553 | 659 | 725 | 7,160 | 63 |
| Rural | 17,012 | 3,746 | 1,711 | 1,310 | 14,828 | 426 |
| **Use of ITN** |  |  |  |  |  |  |
| No | 23,377 | 4,605 | 2,014 | 1,757 | 19,261 | 407 |
| Yes | 3,094 | 694 | 345 | 273 | 2,727 | 75 |
| **Wealth quintile** |  |  |  |  |  |  |
| Least Poor | 5,649 | 771 | 233 | 412 | 4,331 | 21 |
| Second | 5,776 | 886 | 352 | 394 | 4,751 | 55 |
| Third | 5,183 | 1,104 | 519 | 441 | 4,268 | 93 |
| Fourth | 5,006 | 1,210 | 597 | 433 | 4,116 | 174 |
| Most Poor | 4,857 | 1,308 | 669 | 355 | 4,054 | 146 |
| **Total** | **26,471** | **5,299** | **2,370** | **2,035** | **21,988** | **489** |

Table 2 (Table 3 in main text) Number of sampled individuals who used any drugs; used anti-malarials; used non-antimalarials; or parasitological diagnosed before treatment by source among individuals who reported having fever in last two weeks and who took action (N=2,035).

|  | **Use of any drug** | **Use of anti-malarials** | **Use of other drugs, % (95% CI)** | **Parasitological tested for malaria before any treatment** |
| --- | --- | --- | --- | --- |
| **Overall** | 1,935 | 875 | 1,060 | 1,015 |
| **By treatment source** | | | | |
| **Government** |  |  |  |  |
| Hospital | 539 | 241 | 298 | 401 |
| Health centre | 563 | 279 | 284 | 371 |
| Basic health unit | 262 | 123 | 139 | 46 |
| Community Health Worker | 155 | 58 | 97 | 40 |
|  |  |  |  |  |
| **Private** |  |  |  |  |
| Health facility/pharmacy/drug Store | 277 | 133 | 144 | 147 |
| Shop | 10 | 34 | 75 | 5 |
| Other | 30 | 7 | 23 | 4 |

Table 3 (Table 4 in main text) Number of sampled individuals who reported treatment for malaria (N=875) according to the type of anti-malarials used by source.

|  | **Anti-malarial type** | | | | |
| --- | --- | --- | --- | --- | --- |
|  | **AS+SP** | **SP or Chloroquine** | **Quinine** | **Artemether** | **Other** |
| **Overall** | 382 | 122 | 39 | 296 | 36 |
| **By treatment source** |  |  |  |  |  |
| **Government** |  |  |  |  |  |
| Hospital | 114 | 19 | 18 | 80 | 10 |
| Health centre | 128 | 29 | 11 | 105 | 6 |
| Basic health unit | 53 | 21 | 4 | 45 | 0 |
| Community Health Worker | 28 | 10 | 4 | 14 | 2 |
|  |  |  |  |  |  |
| **Private** |  |  |  |  |  |
| Health facility/pharmacy/drug Store | 50 | 23 | 1 | 45 | 14 |
| Shop | 6 | 20 | 1 | 3 | 4 |
| Other | 3 | 0 | 0 | 4 | 0 |
|  |  |  |  |  |  |
| **Overall** |  |  |  |  |  |
